# Structural changes in the sacroiliac joint on MRI and relationship to ASDAS inactive disease in axial spondyloarthritis: a 2-year study comparing treatment with etanercept in EMBARK to a contemporary control cohort in DESIR

**DOI:** 10.1186/s13075-021-02428-8

**Published:** 2021-01-29

**Authors:** Walter P. Maksymowych, Pascal Claudepierre, Manouk de Hooge, Robert G. Lambert, Robert Landewé, Anna Molto, Désirée van der Heijde, Jack F. Bukowski, Heather Jones, Ron Pedersen, Annette Szumski, Bonnie Vlahos, Maxime Dougados

**Affiliations:** 1 Department of Medicine, University of Alberta, 568 Heritage Medical Research Building, Edmonton, AB T6G 2S2 Canada; 2 Universite Paris Est Creteil, EA 7379 – EpidermE, AP-HP, Service de Rhumatologie, Hopital Henri Mondor, Creteil, France; 3 VIB Center of Inflammation Research, Ghent University, Ghent, Belgium; 4 Leiden University Medical Center, Leiden, the Netherlands; 5 Department of Radiology and Diagnostic Imaging, University of Alberta, Edmonton, AB Canada; 6 Amsterdam University Medical Center, loc. Meibergdreef 9 Amsterdam & Zuyderland MC, Heerlen, the Netherlands; 7 René Descartes University, Université de Paris, Department of Rheumatology – Hôpital Cochin, Assistance Publique – Hôpitaux de Paris, INSERM (U1153): Clinical Epidemiology and Biostatistics, PRES Sorbonne Paris-Cité, Paris, France; 8 Global Clinical Affairs, Pfizer, Collegeville, PA USA; 9 Global Medical Affairs, Pfizer, Collegeville, PA USA; 10 Department of Biostatistics, Pfizer, Collegeville, PA USA; 11 Syneos Health, Princeton, NJ USA

**Keywords:** Sacroiliac joint, MRI, Axial spondyloarthritis, Etanercept, Anti-TNF, ASDAS

## Abstract

**Background:**

Limited information is available on the impact of treatment with a tumor necrosis factor inhibitor (TNFi) on structural lesions in patients with recent-onset axial spondyloarthritis (axSpA). We compared 2-year structural lesion changes on magnetic resonance imaging (MRI) in the sacroiliac joints (SIJ) of patients with recent-onset axSpA receiving etanercept in a clinical trial (EMBARK) to similar patients not receiving biologics in a cohort study (DESIR). We also evaluated the relationship between the Ankylosing Spondylitis Disease Activity Score (ASDAS) and change in MRI structural parameters.

**Methods:**

The difference between etanercept (EMBARK) and control (DESIR) in the net percentage of patients with structural lesion change was determined using the SpondyloArthritis Research Consortium of Canada SIJ Structural Score, with and without adjustment for baseline covariates. The relationship between sustained ASDAS inactive disease, defined as the presence of ASDAS < 1.3 for at least 2 consecutive time points 6 months apart, and structural lesion change was evaluated.

**Results:**

This study included 163 patients from the EMBARK trial and 76 from DESIR. The net percentage of patients with erosion decrease was significantly greater for etanercept vs control: unadjusted: 23.9% vs 5.3%; *P* = 0.01, adjusted: 23.1% vs 2.9%; *P* = 0.01. For the patients attaining sustained ASDAS inactive disease on etanercept, erosion decrease was evident in significantly more than erosion increase: 34/104 (32.7%) vs 5/104 (4.8%); *P* < 0.001. A higher proportion had erosion decrease and backfill increase than patients in other ASDAS status categories. However, the trend across ASDAS categories was not significant and decrease in erosion was observed even in patients without a sustained ASDAS response.

**Conclusions:**

These data show that a greater proportion of patients achieved regression of erosion with versus without etanercept. However, the link between achieving sustained ASDAS inactive disease and structural lesion change on MRI could not be clearly established.

**Trial registration:**

EMBARK: ClinicalTrials.gov identifier: NCT01258738, Registered 13 December 2010; DESIR: ClinicalTrials.gov identifier: NCT01648907, Registered 24 July 2012.

**Supplementary Information:**

The online version contains supplementary material available at 10.1186/s13075-021-02428-8.

## Background

The benefit of tumor necrosis factor inhibitor (TNFi) treatment on clinical and magnetic resonance imaging (MRI) features of inflammation in the spine and sacroiliac joints (SIJ) is well documented in patients with ankylosing spondylitis (AS) and non-radiographic axial spondyloarthritis (nr-axSpA) [1–3]. There is less information on the impact on structural lesions, especially in early axSpA when SIJ lesions may not be evident on plain radiography. Recent studies have shown that T1-weighted (T1W) MRI is more sensitive than radiography in detecting SIJ structural lesions, especially erosions [4]. Moreover, MRI reveals additional lesions not observed on plain radiography, namely, fat metaplasia and backfill, which appear in subchondral bone marrow and at erosion sites, respectively, after inflammation resolution and may indicate tissue repair. In an etanercept trial, SIJ structural lesions were already observed in patients with active nr-axSpA and symptom duration < 5 years [5]. SIJ structural lesions routinely evaluated on T1W scans include fat metaplasia, ankylosis, sclerosis, erosion, and fat metaplasia in the erosion cavity [6].

Prospective observation of T1W scans has shown that the morphology of erosions changes as inflammation resolves [7, 8]. During active inflammation, bright signal may be seen in the erosion cavity on fat-suppressed T2-weighted MRI. With resolution of inflammation, increased signal on T1W scans may become evident within the erosion cavity, signifying a reparative process, termed backfill, and the change in MRI appearance resembles transformation of bone marrow edema into fat metaplasia that is often observed following effective treatment [7]. This was observed in a randomized placebo-controlled trial within 12 weeks of starting etanercept [9].

Although treat-to-target is an accepted strategy for achieving sustained clinical remission and preventing structural damage in rheumatoid arthritis (RA), it is unknown whether it will reduce structural damage in axSpA [10]. The Ankylosing Spondylitis Disease Activity Score (ASDAS) has been proposed as an outcome for axSpA; however, the relationship between achieving sustained ASDAS inactive disease (ASDAS < 1.3) [11], and the evolution of structural lesions on MRI is unclear [10]. A prospective cohort demonstrated that change in radiographic sacroiliitis grade over time is associated with change in spinal mobility and function [12].

The objectives of this study were to (1) compare structural lesion changes in the SIJ on T1W MRI in patients with recent-onset axSpA receiving etanercept over 2 years in a clinical trial (Effect of Etanercept on Symptoms and Objective Inflammation in nr-axSpA, a 104-week study [EMBARK]) with similar patients who were TNFi naïve and receiving usual care in an observational study (Devenir des spondyloarthrites indifférenciées récentes [DESIR]); and to (2) evaluate the relationship between sustained ASDAS inactive disease and MRI structural lesions in the SIJ over 2 years. We hypothesized that etanercept would have a greater effect than usual care on decrease in erosion on MRI and that patients with sustained ASDAS inactive disease would be more likely to achieve reduction in structural progression on MRI of the SIJ.

## Patients and methods

The global EMBARK trial has been described previously [13–15]. Briefly, all patients fulfilled the Assessment of SpondyloArthritis international Society (ASAS) criteria for axSpA, but they did not meet the modified New York (mNY) criteria for radiographic axSpA according to central reading. Patients were ≥ 18 and < 50 years old, had experienced symptoms for > 3 months but < 5 years, had a Bath Ankylosing Spondylitis Disease Activity Index (BASDAI) score ≥ 4 out of 10, and had responded inadequately to ≥ 2 nonsteroidal anti-inflammatory drugs. Following 12 weeks of double-blind placebo-controlled therapy, all patients received open-label etanercept 50 mg once weekly for 92 weeks. Patients from the EMBARK trial with available baseline and 104-week MRI data were included in the present analysis.

The French DESIR observational cohort study has also been described previously [16, 17]. Patients were > 18 and < 50 years old and had experienced inflammatory back pain for > 3 months but < 3 years, and rheumatologists diagnosed axSpA based on a probability of ≥ 5 on a 0–10 scale (0 = not confident, 10 = very confident). Patients had no history of treatment with any biologic therapy. Patients from the DESIR cohort were included in this analysis if they had not received any biologic therapy during the first 2 years of follow-up, they met the ASAS criteria for axSpA, and they had baseline and 104-week MRI data available. Per the DESIR study protocol, MRI at 104 weeks was only performed in selected centers in the Paris area.

The studies were conducted in accordance with the International Conference on Harmonization guidelines for Good Clinical Practice and the ethical principles of the Declaration of Helsinki. Prior to study start, institutional review board approval and participant informed consent were obtained. The institutional review board or independent ethics committee at each participating center reviewed and approved the study protocol and consent forms (see “Acknowledgements” for details).

### Structural lesions on MRI

T1W MRI scans of the SIJ at baseline and week 104 from EMBARK and DESIR were anonymized and read per patient by 3 experienced readers who were unaware of image chronology and original patient cohort. The readers independently evaluated the images using the SpondyloArthritis Research Consortium of Canada (SPARCC) SIJ Structural Score (SSS) [6]. Lesion change was based on ≥ 2 of 3 readers measuring change in the same direction; otherwise, it was considered no change. The primary endpoint was the net percentage of patients in the treatment (EMBARK) and control (DESIR) groups with a decrease in erosion, defined as the number of patients with a decrease in score minus the number of patients with an increase in score, divided by the total population. The secondary endpoint was the net percentage of patients in each study group with an increase in backfill. The net percentage of patients with an increase in fat metaplasia and ankylosis was also determined.

The standardized definitions for lesions seen on T1W MRI are provided below [6, 7, 18, 19]:

- Erosion: Full-thickness loss of the dark appearance of iliac or sacral cortical bone, and loss of the normal bright appearance of adjacent bone marrow.
- Backfill: Complete loss of iliac or sacral cortical bone on T1W MRI and increased signal clearly demarcated from adjacent normal marrow by dark signal with irregular contour reflecting sclerosis at the border of the eroded bone.
- Fat metaplasia: Increased signal on T1W MRI with homogeneous signal across the lesion extending > 1 cm in depth from the joint surface.
- Ankylosis: Bone marrow signal on T1W MRI between the sacral and iliac bone marrow with a full-thickness loss of the dark appearance of the iliac and sacral cortical bone.

A score from 0 to 8 per slice for 5 slices was assigned to erosion and fat metaplasia (total score 0–40 for each) [6]. A score from 0 to 4 per slice for 5 slices was assigned to backfill and ankylosis (total score 0–20 for each).

### ASDAS endpoints

We also evaluated the relationship between sustained ASDAS inactive disease and MRI structural parameters using ASDAS measurements taken at months 6, 12, 18, and 24. In both EMBARK and DESIR, all of the components of the ASDAS had to be non-missing in order for ASDAS to be calculated. Responses were assessed sequentially as follows:

1. Was ASDAS < 1.3 present for at least 2 consecutive time points? If yes, then the patient had sustained inactive disease. If no, then:
2. If sustained inactive disease was not achieved, was ASDAS < 2.1 present for at least 2 consecutive time points? If yes, then the patient had sustained low disease activity (LDA). If no, then:
3. The patient was considered to have no sustained response (ASDAS ≥ 2.1 at one visit and same or lower ASDAS at consecutive visit(s)).

### Statistical analysis

All of the analyses were based on observed cases (patients with baseline and week 104 MRI data). Baseline characteristics between study (treatment) cohorts were compared using the Wilcoxon rank-sum test for continuous characteristics and the Mantel-Haenszel *χ*^2^ test for categorical characteristics. Cumulative probability plots were generated to compare the change in structural lesion scores (average change of the 3 readers) between the study cohorts.

The proportion of patients with an increase or decrease in SPARCC SSS for each individual structural lesion was compared within each study cohort. The net percentage of patients with an increase (backfill, fat metaplasia, ankylosis) or decrease (erosion) in score was determined per study cohort and also according to each sustained ASDAS status category. The difference between study cohorts (EMBARK [etanercept] minus DESIR [control]) in the net percentage of patients with an increase or decrease in score was also determined for each structural lesion and according to each sustained ASDAS status category.

These study effects were analyzed without covariates (unadjusted analysis) using one-way analysis of variance (ANOVA) of week 104 structural lesion change categories (change < 0, =0, > 0), and also in analysis of covariance (ANCOVA) models with the following baseline covariates as potential confounders (adjusted analysis): sex, symptom duration, smoking status, human leukocyte antigen (HLA)-B27 status, ASDAS measured using C-reactive protein (CRP), SPARCC MRI SIJ inflammation score, baseline SSS erosion score, and total SIJ score based on the mNY grading system (mNY grade of sacroiliitis for each SIJ is 0–4, total SIJ scoring range is 0–8). For baseline SSS erosion and total SIJ score, the value used was the average of the scores from 3 central readers who independently assessed the MRI scans and radiographs, respectively, blinded to the source cohort.

This analysis was repeated according to sex to determine response differences by sex and by study. Additionally, the effects of study and sustained ASDAS status category on structural lesions were evaluated in ANOVA (unadjusted) and ANCOVA (adjusted) models of change, which included sustained ASDAS status category, study, and their interaction as additional covariates.

We also determined the extent of correlation between the baseline covariates of symptom duration, ASDAS, SPARCC MRI SIJ inflammation score, total SIJ score, and SSS erosion score using Pearson correlations. We conducted two multivariate stepwise regression analyses to determine the best significant subset of predictors for each structural lesion change endpoint. The first analysis forced the predictors of study, sex, and week 104 sustained ASDAS status category into the model, because study and sex were expected to significantly affect outcome, while sustained ASDAS inactive disease was the predictor of interest. The second analysis did not force predictors into the model.

## Results

### Patients

A total of 225 patients were randomized in the EMBARK trial; baseline and 104-week MRI results were available for 163 (72%) patients. A total of 708 patients were enrolled in the DESIR cohort study, 259 (37%) of them were located in Paris and had a 104-week MRI planned per protocol. Of these patients, 155 (60%) fulfilled the ASAS criteria, and among them, 98 (63%) did not receive any biological therapy during the first 2 years of follow-up. Finally, 76 (78%) of these patients received an MRI at 104 weeks. Table 1 presents demographics and baseline characteristics for the patients included in this analysis from EMBARK and DESIR, as well as for the 22 patients from DESIR who would have qualified for inclusion except they did not receive an MRI at week 104.

**Table 1:** Demographics and baseline disease characteristics

	Etanercept (EMBARK) ***N*** = 163^**a**^	Control (DESIR) ***N*** = 76^**a**^	***P*** value	Control (DESIR) excluded^**b**^ ***N*** = 22
Age, y	31.7 (7.9)	31.7 (7.5)	0.94^c^	31.9 (6.7)
Male, n/N (%)	105/163 (64.4)	45/76 (59.2)	0.44^d^	12/22 (54.5)
Symptom duration, years	2.4 (1.8)	1.5 (0.9)	< 0.001^c^	1.5 (0.9)
Current smoker, *n*/*N* (%)	37/163 (22.7)	29/75 (38.7)	0.01^d^	5/22 (22.7)
HLA-B27(+), *n*/*N* (%)	121/157 (77.1)	66/76 (86.8)	0.08^d^	17/22 (77.3)
BASDAI (0–10)	6.0 (1.8)	3.4 (2.0)	< 0.001^c^	4.5 (2.1)
ASDAS	3.0 (1.0)	2.1 (0.9)	< 0.001^c^	2.6 (0.9)
BASFI (0–10 cm VAS)	4.1 (2.4)	2.0 (1.9)	< 0.001^c^	3.1 (2.9)
CRP, mg/L	7.4 (11.5)	6.0 (8.1)	0.19^c^	7.1 (13.0)
Erosion SSS (0–40)^f^	2.2 (3.0)	2.8 (3.5)	0.12^c^	0.6 (1.5), 2.4 (4.7), 1.5 (2.6)
Backfill SSS (0–20)	0.5 (1.3)	0.3 (0.7)	0.50^c^	NA
Fat metaplasia SSS (0–40)^f^	0.6 (1.7)	0.5 (1.8)	0.91^c^	1.5 (3.2), 1.8 (3.7), 1.7 (3.9)
Ankylosis SSS (0–20)^f^	0.3 (1.3)	0.2 (1.1)	0.11^c^	0.1 (0.6), 0.1 (0.3), 0.2 (0.7)
SPARCC MRI SIJ inflammation score (0–72)^f^	8.5 (11.0)	6.0 (9.4)	0.003^c^	4.5 (6.5), 3.3 (4.0), 2.7 (3.9)
SPARCC MRI SIJ inflammation score ≥ 2, *n*/*N* (%)^f^	99/161 (61.5)	33/76 (43.4)	0.009^d^	11/20 (55.0), 9/19 (47.4), 9/21 (42.9)
Total SIJ score (mNY grade 0–8)^f^	1.5 (1.3)	1.6 (1.6)	0.76^c^	1.5 (2.2), 1.4 (2.0), 1.7 (1.6)
SIJ radiograph met mNY criteria (%)^e, f^	19/156 (12.2)	10/68 (14.7)	0.61^d^	7/22 (31.8), 5/20 (25.0), 4/21 (19.0)

^a^Patients with week 104 MRI data

^b^Not included in the analysis because a week 104 MRI was not available. Included in the table for comparison only

^c^Wilcoxon rank-sum

^d^Mantel-Haenszel

^e^Radiographs from EMBARK and DESIR cohorts were assessed by 3 central readers who were not the same readers that conducted assessment of radiographs at screening for inclusion in the EMBARK trial

^f^For the patients from DESIR who were excluded from this analysis, we have included 3 scores because there were 3 sets of central readers in 3 independent reading campaigns

Values are mean (standard deviation) unless otherwise noted

*ASDAS* Ankylosing Spondylitis Disease Activity Score, *BASDAI* Bath Ankylosing Spondylitis Disease Activity Index, *BASFI* Bath Ankylosing Spondylitis Functional Index, *CRP* C-reactive protein, *HLA* human leukocyte antigen, *mNY* modified New York, *NA* not available, *SPARCC MRI SIJ inflammation score* Spondyloarthritis Research Consortium of Canada Magnetic Resonance Imaging Sacroiliac Joint score (a score of MRI bone marrow edema), *SSS* SIJ Structural Score, *VAS* visual analog scale

### Baseline characteristics

At baseline, symptom duration was significantly longer in the etanercept group, and function, as measured by the Bath Ankylosing Spondylitis Functional Index (BASFI), was significantly worse. The disease activity markers of BASDAI, ASDAS, and SPARCC MRI SIJ inflammation were significantly higher in the etanercept group; the control group included a higher proportion of smokers. Radiographic SIJ damage and the SPARCC structural scores for each lesion did not differ significantly between the two groups. The proportions of patients in the etanercept vs control group, respectively, with baseline structural scores of 0 were 55.2% vs 53.9% for erosion, 87.1% vs 93.4% for backfill, 90.2% vs 94.7% for fat metaplasia, and 96.9% vs 94.7% for ankylosis; these differences were not significant.

Most disease characteristics were similar between the patients from DESIR who were included and excluded from the analysis. The excluded patients had higher fat metaplasia structural scores and a greater percentage of patients with SIJ radiographs that met mNY criteria.

### Net percentage of patients with change in structural lesions

**Fig. 1:**
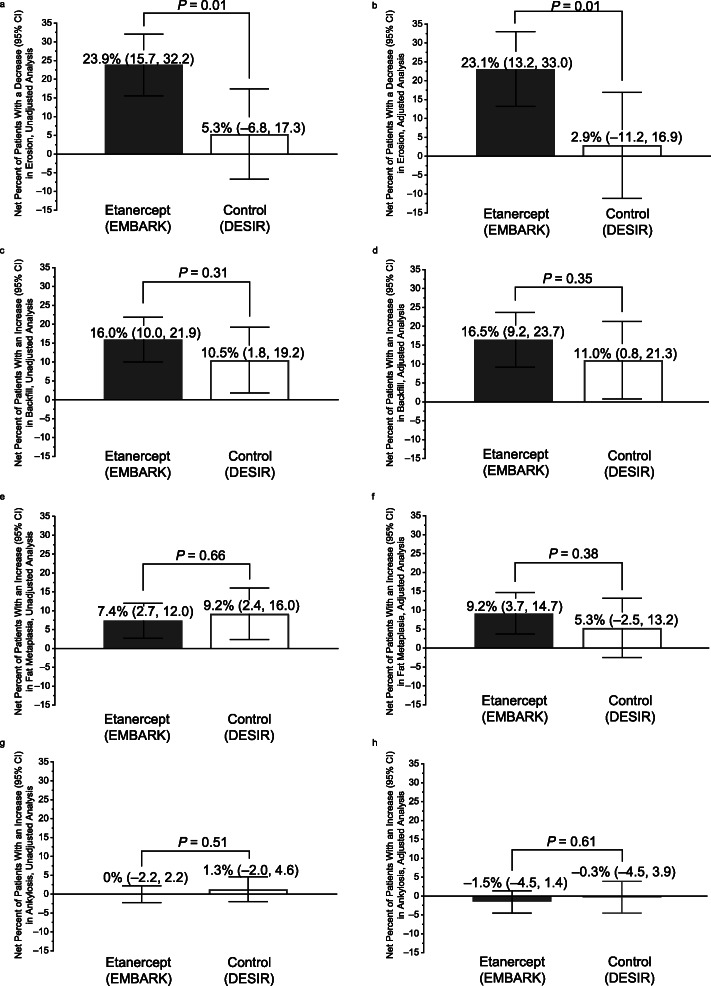
Net percentage of patients with axial spondyloarthritis with decrease in erosion, unadjusted (**a**) and adjusted analysis (**b**); increase in backfill, unadjusted (**c**) and adjusted analysis (**d**), increase in fat metaplasia, unadjusted (**e**) and adjusted analysis (**f**), increase in ankylosis, unadjusted (**g**) and adjusted analysis (**h**), baseline to week 104

For the etanercept group, erosion mean (95% CI) change was − 0.81 (− 1.09, − 0.53) and median (Q1, Q3) change was 0 (− 1.3, 0); for the control group, erosion mean change was − 0.23 (− 0.64, 0.18) and median change was 0 (− 0.33, 0.33). The net percentage (95% CI) of patients with a decrease in erosion was significantly greater for etanercept vs control in the unadjusted analysis: 23.9% (15.7, 32.2) vs 5.3% (− 6.8, 17.3), respectively; *P* = 0.01. The results of the adjusted analysis were very similar (Fig. 1a, b).

For the etanercept group, backfill mean (95% CI) change was 0.62 (0.37, 0.87) and median (Q1, Q3) change was 0 (0, 0.33); for the control group, backfill mean change was 0.54 (0.18, 0.91) and median change was 0 (0, 0.17). The net percentage of patients with an increase in backfill was higher for etanercept vs control in both analyses; however, the difference was not statistically significant. The results for the unadjusted analysis were 16.0% (10.0, 21.9) and 10.5% (1.8, 19.2) for etanercept and control, respectively; *P* = 0.31. The results for the adjusted analysis were very similar (Fig. 1c, d).

The net percentage of patients with an increase in fat metaplasia was higher for etanercept vs control in the adjusted analysis only; the difference was not statistically significant (Fig. 1e, f). Differences between the groups in ankylosis were slight (Fig. 1g, h). Additional file 1, Table S1 presents the absolute numbers, proportions, and net percentages of patients with an increase or decrease in each structural lesion at week 104.

**Fig. 2:**
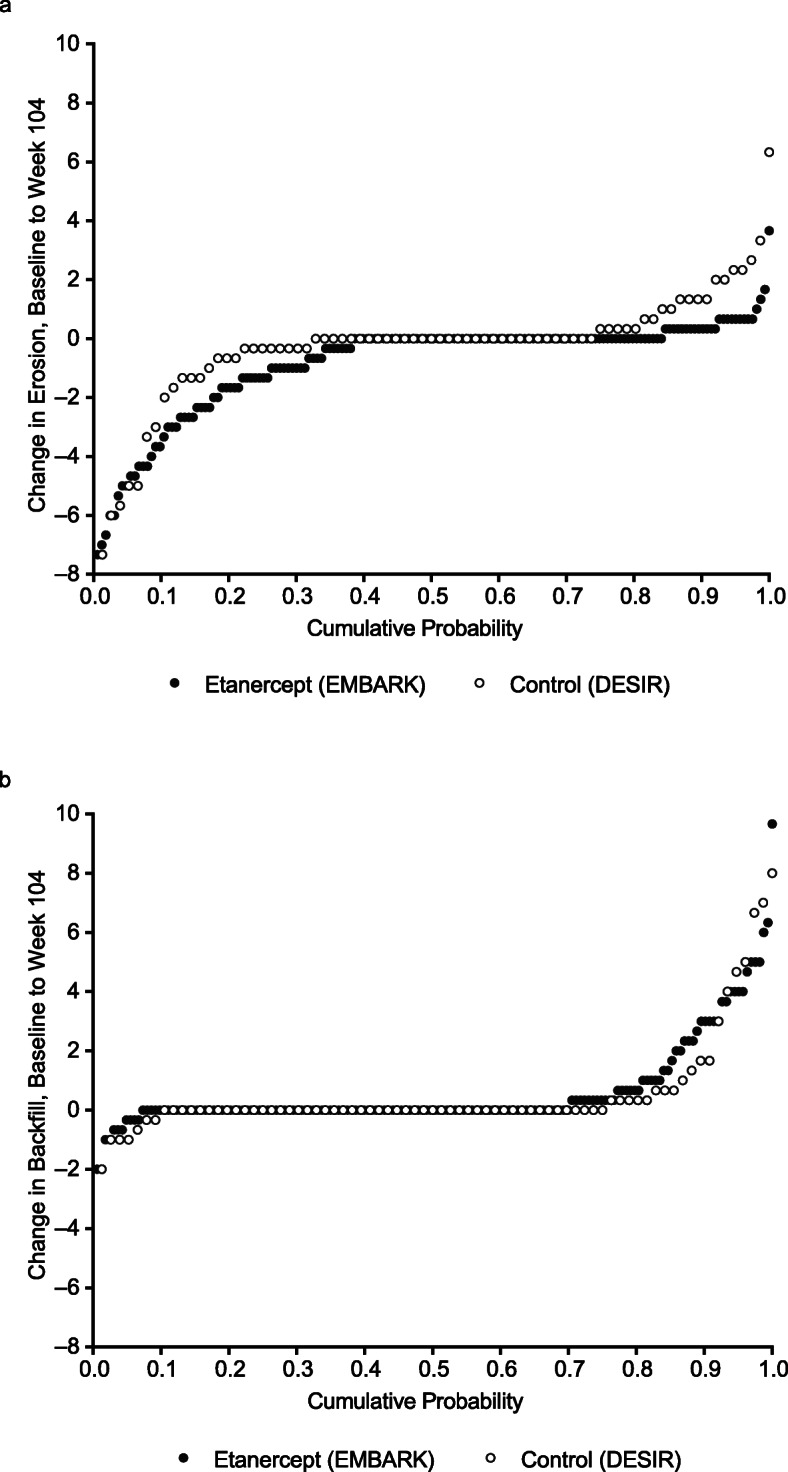
Cumulative probability of change in MRI structural lesion score in patients with axial spondyloarthritis for erosion (**a**) and backfill (**b**) over 104 weeks, average of the readers. *MRI* magnetic resonance imaging

In the cumulative probability plots for change in MRI structural lesion score, the etanercept group showed a greater trend than the control group toward erosion decrease, backfill increase (Fig. 2), and fat metaplasia increase (Additional file 1, Figure S1). There was little change in ankylosis in either group.

### Pearson correlations and multivariate stepwise regression analyses

The Pearson correlations demonstrated significant relationships between the baseline covariates of ASDAS and SPARCC MRI SIJ inflammation score (*r* = 0.17, *P* < 0.05), SPARCC MRI SIJ inflammation score and total SIJ score (*r* = 0.26, *P* < 0.001), SPARCC MRI SIJ inflammation score and SSS erosion score (*r* = 0.64, *P* < 0.001), and total SIJ score and SSS erosion score (*r* = 0.45, *P* < 0.001) (Additional file 1, Table S2).

The multivariate stepwise regression analysis with forced predictors determined that the best significant subset of predictors of week 104 structural lesion change was baseline SSS erosion score and baseline SPARCC MRI SIJ inflammation score; both were predictors of change in all lesions except ankylosis (Additional file 1, Table S3). Sustained ASDAS < 1.3 was not a significant predictor of structural lesion change. The results were similar in the analysis without forced predictors (Additional file 1, Table S4).

### Analysis of structural lesion change according to sex

The occurrence of erosion decrease, backfill increase, and fat metaplasia increase was greater for males than females in both studies (Table 2). This resulted in a significant and higher net percentage of erosion decrease, backfill increase, and fat metaplasia increase for males than for females, especially in the etanercept group. For erosion, the net percentage of males vs females with a decrease was 31.4% vs 10.3%, respectively, for etanercept, and 8.9% vs 0% for control. At baseline, the median SSS erosion score in the etanercept group was 1.7 for males and 0 for females; *P* < 0.001, and in the control group it was 1.7 for males and 1.0 for females; the difference was not statistically significant. The net percentage of males vs females with an increase in backfill was 21.9% vs 5.2%, respectively, for etanercept, and 13.3% vs 6.5% for control, and for fat metaplasia, it was 10.5% vs 1.7%, respectively, for etanercept, and 13.3% vs 3.2% for control.

**Table 2:** Structural lesion change according to sex in patients with axial spondyloarthritis, baseline to week 104

Structural lesion	Study cohort	Sex	Lesion decreased *n*/*N* (%)	Lesion increased *n*/*N* (%)	Net % patients with decrease within study cohort (95% CI)	*P* value for lesion decrease vs increase	*P* value for difference in sex (both studies pooled)	*P* value for difference in study (both sexes pooled)	*P* value for difference in sex (by study)	*P* value for difference in study (by sex)
Erosion	Etanercept (EMBARK)	Male	39/105 (37.1)	6/105 (5.7)	31.4% (21.3, 41.6)	< 0.001	0.048	0.03	0.02	0.02
		Female	7/58 (12.1)	1/58 (1.7)	10.3% (− 3.3, 24.0)	0.14				0.38
	Control (DESIR)	Male	10/45 (22.2)	6/45 (13.3)	8.9% (− 6.6, 24.4)	0.26			0.47	
		Female	4/31 (12.9)	4/31 (12.9)	0% (− 18.7, 18.7)	1.0				
Structural lesion	Study cohort		Lesion increased n/N (%)	Lesion decreased n/N (%)	Net % patients with increase within study cohort (95% CI)	*P* value for lesion increase vs decrease	*P* value for difference in sex (both studies pooled)	*P* value for difference in study (both sexes pooled)	*P* value for difference in sex (by study)	*P* value for difference in study (by sex)
Backfill	Etanercept (EMBARK)	Male	24/105 (22.9)	1/105 (1.0)	21.9% (14.6, 29.2)	< 0.001	0.03	0.50	0.008	0.21
		Female	3/58 (5.2)	0/58 (0)	5.2% (− 4.6, 15.0)	0.30				0.88
	Control (DESIR)	Male	8/45 (17.8)	2/45 (4.4)	13.3% (2.2, 24.5)	0.02			0.44	
		Female	2/31 (6.5)	0/31 (0)	6.5% (− 7.0, 19.9)	0.35				
Fat metaplasia	Etanercept (EMBARK)	Male	13/105 (12.4)	2/105 (1.9)	10.5% (4.7, 16.2)	< 0.001	0.03	0.61	0.07	0.59
		Female	1/58 (1.7)	0/58 (0)	1.7% (− 6.0, 9.5)	0.66				0.82
	Control (DESIR)	Male	6/45 (13.3)	0/45 (0)	13.3% (4.6, 22.1)	0.003			0.15	
		Female	1/31 (3.2)	0/31 (0)	3.2% (− 7.4, 13.8)	0.55				
Ankylosis	Etanercept (EMBARK)	Male	2/105 (1.9)	1/105 (1.0)	1.0% (− 1.8, 3.7)	0.50	0.24	0.47	0.26	0.62
		Female	0/58 (0)	1/58 (1.7)	− 1.7% (− 5.5, 2.0)	0.37				0.59
	Control (DESIR)	Male	1/45 (2.2)	0/45 (0)	2.2% (− 2.0, 6.5)	0.31			0.51	
		Female	0/31 (0)	0/31 (0)	0% (− 5.1, 5.1)	1.0				

### Change in structural lesions according to sustained ASDAS status

The analysis according to sustained ASDAS status included 161 and 76 patients from the EMBARK and DESIR cohorts, respectively. Additional file 1: Table S5 presents the absolute numbers, proportions, and net percentages of patients with an increase or decrease in erosion or backfill between baseline and week 104, according to sustained ASDAS status.

#### Erosion

**Fig. 3:**
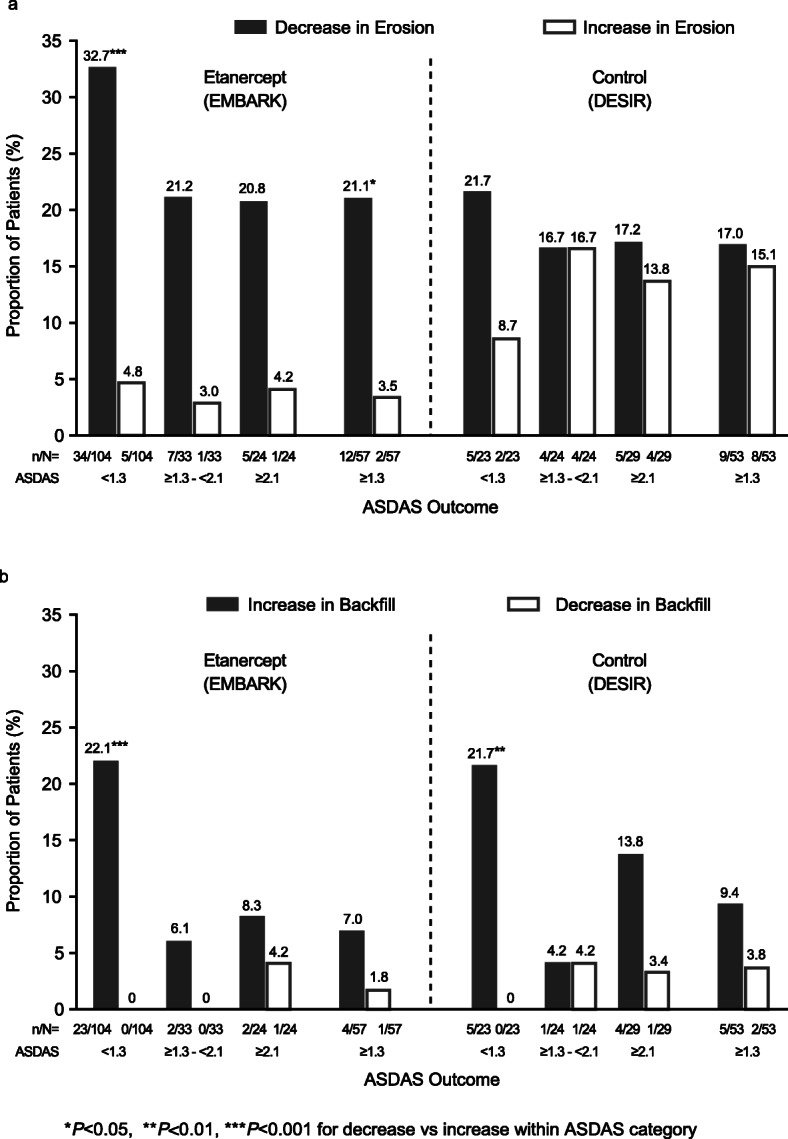
Proportion of patients with axial spondyloarthritis with decrease or increase in erosion (**a**), increase or decrease in backfill (**b**) according to ASDAS outcome, baseline to week 104. *ASDAS* Ankylosing Spondylitis Disease Activity Score

**Fig. 4:**
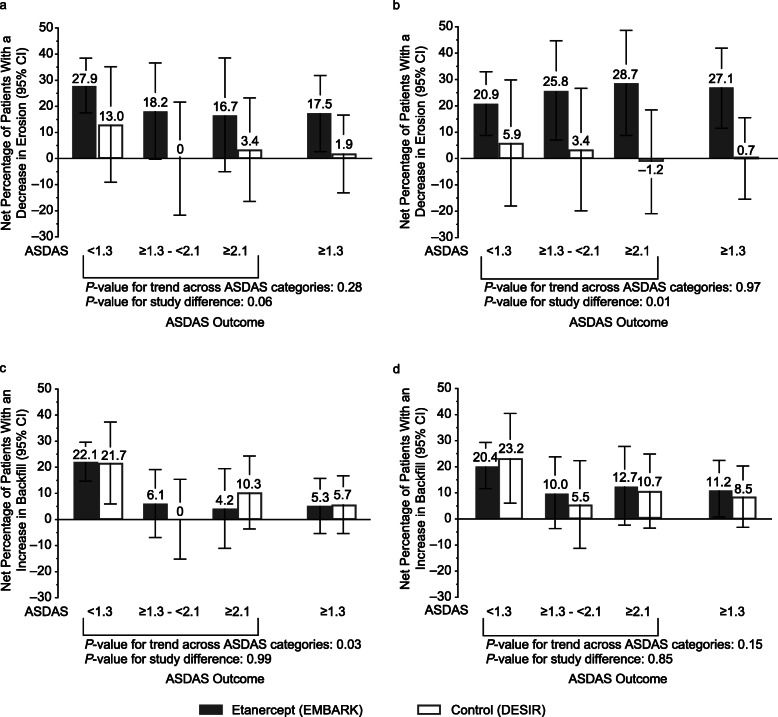
Net percentage of patients with axial spondyloarthritis with decrease in erosion, unadjusted (**a**) and adjusted (**b**), and increase in backfill, unadjusted (**c**) and adjusted (**d**) according to ASDAS outcome, baseline to week 104. *ASDAS* Ankylosing Spondylitis Disease Activity Score

For the etanercept group, values for mean (95% CI) change in erosion for the sustained ASDAS response categories of < 1.3, ≥1.3 to < 2.1, and ≥ 2.1 were − 1.0 (− 1.4, − 0.63), − 0.55 (− 1.4, 0.05), and − 0.38 (− 1.1, 0.30), respectively. For the control group, the corresponding values were − 0.55 (− 1.4, 0.25), −0.01 (−0.71, 0.68), and − 0.15 (−0.76, 0.46). For the patients in the etanercept group with sustained ASDAS inactive disease, erosion decrease was evident in significantly more patients than erosion increase: 34/104 (32.7%) vs 5/104 (4.8%); *P* < 0.001 (Fig. 3a). Erosion decrease was also evident in significantly more patients than erosion increase in the etanercept group with ASDAS ≥ 1.3 (Fig. 3a), even though there was no significant trend across the 3 ASDAS categories (Fig. 4a, b). The highest percentage of patients with erosion decrease was in the sustained ASDAS inactive disease category. Additionally, the percentage of patients with erosion increase was low for all 3 ASDAS categories. In the control group, there was no significant difference between patients with a decrease and increase in erosion for any of the ASDAS outcomes (Fig. 3a). The difference between the study groups in the net percentage of patients with a decrease in erosion according to ASDAS status category was significant in the adjusted analysis (*P* = 0.01) (Fig. 4b).

#### Backfill

For the etanercept group, values for mean change (95% CI) in backfill for the sustained ASDAS response categories of < 1.3, ≥ 1.3 to < 2.1, and ≥ 2.1 were 0.84 (0.50, 1.2), 0.41 (− 0.13, 0.95), and 0.03 (− 0.51, 0.57), respectively. For the control group, the corresponding values were 0.90 (0.18, 1.6), 0.10 (−0.54, 0.73), and 0.63 (0.14, 1.1). In both study groups, an increase in backfill was evident in significantly more patients than a decrease in backfill only in those patients with sustained ASDAS inactive disease: 23/104 (22.1%) vs 0/104 (0%), respectively, for etanercept; *P* < 0.001, and 5/23 (21.7%) vs 0/23 (0%), for control; *P* = 0.007 (Fig. 3b). The net percentage of patients with an increase in backfill was not significant for the other ASDAS outcomes in either study group (Additional file 1, Table S5). The trend across ASDAS status categories was significant in the unadjusted analysis (*P* = 0.03) (Fig. 4c), but not in the adjusted analysis (Fig. 4d). Increases and decreases in backfill were similar between both study groups across all ASDAS categories.

#### Fat metaplasia and ankylosis

In the etanercept group, an increase in fat metaplasia was evident in significantly more patients than a decrease in fat metaplasia in those patients with sustained ASDAS inactive disease: 11/104 (10.6%) vs 2/104 (1.9%), respectively; *P* = 0.004 (Additional file 1, Figure S2). In the control group, an increase in fat metaplasia was evident in significantly more patients than a decrease in those patients with sustained LDA (ASDAS ≥ 1.3 to < 2.1) but not inactive disease: 3/24 (12.5%) vs 0/24 (0%), respectively; *P* = 0.045. The trend across ASDAS status categories was not significant. Few patients in either group experienced a change in ankylosis (Additional file 1, Figure S2).

## Discussion

Our data support the hypothesis that etanercept has more effect than usual care on SIJ erosion development, with significantly more patients demonstrating decreased erosion in unadjusted analyses and in analyses adjusted for baseline differences in symptom duration, disease activity, MRI erosion, and radiographic SIJ grading. This corresponds with an EMBARK study analysis in which patients receiving etanercept had a significantly greater reduction in erosion and increase in backfill than those receiving placebo after 12 weeks of therapy [9].

The results less clearly support the hypothesis that attaining sustained ASDAS inactive disease is relevant to the amelioration of erosion. In patients who did achieve this status, a decrease in erosion was evident in significantly more patients than an increase in erosion only in the etanercept group, while an increase in backfill was evident in significantly more patients than a decrease in backfill in both study groups.

MRI permits a more precise assessment of individual structural lesions than plain radiography and this study has provided further evidence that erosive lesions may not progress and may even decrease after effective anti-inflammatory therapy [20]. Our understanding of tissue repair following inflammation in axSpA is evolving; prospective studies using MRI have provided valuable insights [15, 21]. The first descriptions of SIJ erosion on MRI of patients with axSpA were based on cross-sectional observation of a breach in subchondral bone and loss of adjacent marrow matrix, seen as hypointense signal on T1W MRI and bright signal on fat-suppressed MRI [22]. Additional cross-sectional data have reported high T1W signal in the SIJ space with 96% specificity for axSpA [23]. Recent prospective evaluation of MRI structural lesions has linked these observations by demonstrating that the erosion appearance changes as inflammation resolves. High T1W signal becomes evident in the cavity of the erosion, reflecting new reparative tissue that has replaced inflammatory tissue in the erosion (backfill) [7, 8]. Moreover, data from randomized placebo-controlled trials show that transformation of the erosion appearance may be observed within 12 weeks after initiating TNFi treatment [9, 21]. While this erosion change is well documented on MRI, this evolution may also be observed in patients receiving usual care, as in DESIR, if inactive disease is sustained. In a previous trial comparing naproxen and placebo versus infliximab and naproxen in patients with axSpA symptoms for ≤ 3 years, this evolution was also observed in patients who received naproxen alone [24].

The evolution of erosion to backfill may not be observed in all patients, and erosions may decrease in some patients in the absence of this tissue response. The factors that influence the development of this tissue response following resolution of inflammation are unclear, though our data suggest that gender may play a role. Also, the extent of erosion may decrease in very early disease as the repair process re-establishes a more normal appearing joint surface. This is in contrast to patients with later disease when bone formation leading to ankylosis will also result in less evidence of erosion, although significant progression to ankylosis was not observed in either cohort.

Treat-to-target recommendations in axSpA stress disease activity monitoring using the ASDAS, since a longitudinal relationship between ASDAS level and radiographic progression in the spine has been reported in axSpA [10]. Accordingly, recommendations that clinicians target attainment of ASDAS inactive disease in patients with axSpA are analogous to the treat-to-target concept in RA and other chronic disorders [10]. However, acceptance in clinical practice requires demonstration of a relationship between ASDAS inactive disease and amelioration of structural progression endpoints on imaging. Moreover, randomized trials should demonstrate that using the ASDAS in a treat-to-target strategy results in improved structural damage endpoints versus usual care.

Our data provide limited evidence to support this concept by demonstrating that sustained ASDAS inactive disease was associated with decreased erosion on MRI and increased reparation in the etanercept group. However, our data did not demonstrate a significant trend in decreased erosion across several ASDAS status categories. The decrease in erosion was less evident in the control group, and the difference between etanercept and control was statistically significant in the adjusted analysis. Of note, amelioration of erosion, rather than increased erosion, was evident in all ASDAS status categories for etanercept, including in patients with persistent disease activity (ASDAS ≥ 2.1). This may reflect an impact of etanercept on disease activity parameters not captured by the ASDAS and/or an effect on bone resorption that is uncoupled from inflammation. TNF is a major regulatory cytokine for osteoclastic activation and such uncoupling between effects on bone erosion and clinical parameters of inflammation has been reported in RA [25]. The clinical and prognostic significance of MRI erosion as a relevant structural damage endpoint for evaluating treat-to-target strategies in axSpA requires further study.

A study weakness is that it was not prospectively randomized and controlled; the treatment group was compared with a contemporary cohort from an observational study. Symptom duration was longer and disease activity markers were higher in EMBARK; this was not surprising since those patients were eligible for TNFi initiation. We adjusted for covariates that may affect radiographic progression; however, statistical adjustment does not entirely correct for baseline differences. Additionally, the control group was substantially smaller than the treatment group; therefore, we hesitate to draw firm conclusions from these data.

We chose the covariates based on our knowledge of clinical and laboratory variables associated with radiographic lesion development [17, 26, 27]. However, since our knowledge of independent factors associated with development of SIJ structural lesions on MRI is incomplete, this is a study limitation. A published multivariate analysis of a prospective cohort demonstrated that only change in SPARCC MRI SIJ inflammation and baseline SSS erosion score were independently associated with change in MRI SSS erosion score [8]. Clinical measures of disease activity, demographics, and HLA-B27 were not associated with development of MRI structural features [8]. Our multivariate stepwise regression analyses confirmed these associations. Additionally, we found that for erosion, backfill, and fat metaplasia, the response was greater for males than females. The published literature has noted a greater treatment response for males compared to females with axSpA [28–30].

## Conclusions

This study demonstrated that a higher proportion of patients achieved regression of erosion with versus without etanercept. This effect of etanercept was observed across all sustained ASDAS status categories. The clinical relevance of this change in MRI erosion and backfill in the SIJ and the relationship to future development of ankylosis in the spine requires further study.

## Supplementary Information

**Additional file 1: Table S1.** Lesion change on MRI in patients with axial spondyloarthritis, baseline to Week 104; **Figure S1.** Cumulative probability of change in MRI structural lesion score in patients with axial spondyloarthritis for fat metaplasia (a) and ankylosis (b) over 104 weeks, average of the readers; **Table S2.** Pearson correlations between the baseline covariates; **Table S3.** Significant subset of predictors of Week 104 structural lesion change categories, from stepwise selection models (with predictors of study, sex, and Week 104 3-level ASDAS forced into model); **Table S4.** Significant subset of predictors of Week 104 structural lesion change categories, from stepwise selection models (with no forcing of predictors into model); **Table S5.** Decrease or increase in MRI structural lesions of erosion and backfill according to sustained ASDAS outcome in patients with axial spondyloarthritis, baseline to Week 104; **Figure S2.** Proportion of patients with axial spondyloarthritis with increase or decrease in fat metaplasia (a), and increase or decrease in ankylosis (b) according to ASDAS outcome, baseline to Week 104.

## Data Availability

Upon request, and subject to certain criteria, conditions, and exceptions (see https://www.pfizer.com/science/clinical-trials/trial-data-and-results for more information), Pfizer will provide access to individual de-identified participant data from Pfizer-sponsored global interventional clinical studies conducted for medicines, vaccines, and medical devices (1) for indications that have been approved in the US and/or EU or (2) in programs that have been terminated (i.e., development for all indications has been discontinued). Pfizer will also consider requests for the protocol, data dictionary, and statistical analysis plan. Data may be requested from Pfizer trials 24 months after study completion. The de-identified participant data will be made available to researchers whose proposals meet the research criteria and other conditions, and for which an exception does not apply, via a secure portal. To gain access, data requestors must enter into a data access agreement with Pfizer.

## Abbreviations

ANCOVA: Analysis of covariance
ANOVA: Analysis of variance
AS: Ankylosing spondylitis
ASAS: Assessment of SpondyloArthritis international Society
ASDAS: Ankylosing Spondylitis Disease Activity Score
axSpA: Axial spondyloarthritis
BASDAI: Bath Ankylosing Spondylitis Disease Activity Index
BASFI: Bath Ankylosing Spondylitis Functional Index
CRP: C-reactive protein
HLA: Human leukocyte antigen
LDA: Low disease activity
mNY: Modified New York
MRI: Magnetic resonance imaging
nr-axSpA: Non-radiographic axial spondyloarthritis
RA: Rheumatoid arthritis
SIJ: Sacroiliac joint
SPARCC: SpondyloArthritis Research Consortium of Canada
SSS: Sacroiliac joint Structural Score
T1W: T1-weighted
TNFi: Tumor necrosis factor inhibitor
VAS: Visual analog scale

## References

[1] Sepriano A, Regel A, van der Heijde D, Braun J, Baraliakos X, Landewé R (2017). Efficacy and safety of biological and targeted-synthetic DMARDs: a systematic literature review informing the 2016 update of the ASAS/EULAR recommendations for the management of axial spondyloarthritis. RMD Open.

[2] Callhoff J, Sieper J, Weiß A, Zink A, Listing J (2015). Efficacy of TNFα blockers in patients with ankylosing spondylitis and non-radiographic axial spondyloarthritis: a meta-analysis. Ann Rheum Dis.

[3] Corbett M, Soares M, Jhuti G, Rice S, Spackman E, Sideris E (2016). Tumour necrosis factor-α inhibitors for ankylosing spondylitis and non-radiographic axial spondyloarthritis: a systematic review and economic evaluation. Health Technol Assess.

[4] Diekhoff T, Hermann K-GA, Greese J, Schwenke C, Poddubnyy D, Hamm B (2017). Comparison of MRI with radiography for detecting structural lesions of the sacroiliac joint using CT as standard of reference: results from the SIMACT study. Ann Rheum Dis.

[5] Maksymowych WP, Wichuk S, Dougados M, Jones H, Szumski A, Bukowski JF (2017). MRI evidence of structural changes in the sacroiliac joints of patients with non-radiographic axial spondyloarthritis even in the absence of MRI inflammation. Arthritis Res Ther..

[6] Maksymowych WP, Wichuk S, Chiowchanwisawakit P, Lambert RG, Pedersen SJ (2015). Development and preliminary validation of the spondyloarthritis research consortium of Canada magnetic resonance imaging sacroiliac joint structural score. J Rheumatol.

[7] Maksymowych WP, Wichuk S, Chiowchanwisawakit P, Lambert RG, Pedersen SJ (2014). Fat metaplasia and backfill are key intermediaries in the development of sacroiliac joint ankylosis in patients with ankylosing spondylitis. Arthritis Rheum.

[8] Pedersen SJ, Wichuk S, Chiowchanwisawakit P, Lambert RG, Maksymowych WP (2014). Tumor necrosis factor inhibitor therapy but not standard therapy is associated with resolution of erosion in the sacroiliac joints of patients with axial spondyloarthritis. Arthritis Res Ther.

[9] Maksymowych WP, Wichuk S, Dougados M, Jones H, Pedersen R, Szumski A (2018). Modification of structural lesions on magnetic resonance imaging of the sacroiliac joints by etanercept in the EMBARK trial: a 12-week randomised placebo-controlled trial in patients with non-radiographic axial spondyloarthritis. Ann Rheum Dis.

[10] Smolen JS, Schöls M, Braun J, Dougados M, FitzGerald O, Gladman DD (2018). Treating axial spondyloarthritis and peripheral spondyloarthritis, especially psoriatic arthritis, to target: 2017 update of recommendations by an international task force. Ann Rheum Dis.

[11] Machado PM, Landewé R, van der Heijde D. Ankylosing Spondylitis Disease Activity Score (ASDAS): 2018 update of the nomenclature for disease activity states. Ann Rheum Dis. 2018: Epub ahead of print: [11 September 2018]; 10.1136/annrheumdis-2018-213184. 10.1136/annrheumdis-2018-213184 29453216

[12] Protopopov M, Sieper J, Haibel H, Listing J, Rudwaleit M, Poddubnyy D (2017). Relevance of structural damage in the sacroiliac joints for the functional status and spinal mobility in patients with axial spondyloarthritis: results from the German Spondyloarthritis Inception Cohort. Arthritis Res Ther..

[13] Dougados M, van der Heijde D, Sieper J, Braun J, Maksymowych WP, Citera G (2014). Symptomatic efficacy of etanercept and its effects on objective signs of inflammation in early nonradiographic axial spondyloarthritis: a multicenter, randomized, double-blind, placebo-controlled trial. Arthritis Rheum.

[14] Maksymowych WP, Dougados M, van der Heijde D, Sieper J, Braun J, Citera G (2016). Clinical and MRI responses to etanercept in early non-radiographic axial spondyloarthritis: 48-week results from the EMBARK study. Ann Rheum Dis.

[15] Dougados M, van der Heijde D, Sieper J, Braun J, Citera G, Lenaerts J (2017). Effects of long-term etanercept treatment on clinical outcomes and objective signs of inflammation in early non-radiographic axial spondyloarthritis: 104-week results from the EMBARK study. Arthritis Care Res.

[16] Dougados M, Etcheto A, Molto A, Alonso S, Bouvet S, Daurès J-P (2015). Clinical presentation of patients suffering from recent onset chronic inflammatory back pain suggestive of spondyloarthritis: the DESIR cohort. Joint Bone Spine.

[17] Dougados M, Demattei C, van den Berg R, Vo Hoang V, Thevenin F, Reijnierse M (2016). Rate and predisposing factors for sacroiliac joint radiographic progression after a two-year follow-up period in recent-onset spondyloarthritis. Arthritis Rheumatol.

[18] Weber U, Pedersen SJ, Østergaard M, Rufibach K, Lambert RGW, Maksymowych WP (2012). Can erosions on MRI of the sacroiliac joints be reliably detected in patients with ankylosing spondylitis? - a cross-sectional study. Arthritis Res Ther.

[19] Østergaard M, Maksymowych WP, Pedersen SJ, Chiowchanwisawakit P, Lambert RGW (2009). Structural lesions detected by magnetic resonance imaging in the spine of patients with spondyloarthritis – definitions, assessment system, and reference image set. J Rheumatol.

[20] Dougados M, Maksymowych WP, Landewé RBM, Moltó A, Claudepierre P, de Hooge M (2018). Evaluation of the change in structural radiographic sacroiliac joint damage after 2 years of etanercept therapy (EMBARK trial) in comparison to a contemporary control cohort (DESIR cohort) in recent onset axial spondyloarthritis. Ann Rheum Dis.

[21] Pedersen SJ, Poddubnyy D, Sørensen IJ, Loft A-G, Hindrup JS, Thamsborg G (2016). Course of magnetic resonance imaging–detected inflammation and structural lesions in the sacroiliac joints of patients in the randomized, double-blind, placebo-controlled Danish multicenter study of adalimumab in spondyloarthritis, as assessed by the Berlin and Spondyloarthritis Research Consortium of Canada methods. Arthritis Rheumatol..

[22] Rudwaleit M, Jurik AG, Hermann K-GA, Landewé R, van der Heijde D, Baraliakos X (2009). Defining active sacroiliitis on magnetic resonance imaging (MRI) for classification of axial spondyloarthritis: a consensual approach by the ASAS/OMERACT MRI group. Ann Rheum Dis.

[23] Laloo F, Herregods N, Varkas G, Jaremko J, Baraliakos X, Elewaut D (2017). MR signal in the sacroiliac joint space in spondyloarthritis: a new sign. Eur Radiol.

[24] Poddubnyy D, Listing J, Sieper J (2016). Brief report: course of active inflammatory and fatty lesions in patients with early axial spondyloarthritis treated with infliximab plus naproxen as compared to naproxen alone: results from the infliximab as first line therapy in patients with early active axial spondyloarthritis trial. Arthritis Rheumatol.

[25] van den Berg WB, van Riel PLCM (2005). Uncoupling of inflammation and destruction in rheumatoid arthritis: myth or reality?. Arthritis Rheum.

[26] Poddubnyy D, Rudwaleit M, Haibel H, Listing J, Märker-Hermann E, Zeidler H (2011). Rates and predictors of radiographic sacroiliitis progression over 2 years in patients with axial spondyloarthritis. Ann Rheum Dis.

[27] Poddubnyy D, Sieper J (2014). Similarities and differences between nonradiographic and radiographic axial spondyloarthritis: a clinical, epidemiological and therapeutic assessment. Curr Opin Rheumatol.

[28] Gremese E, Bernardi S, Bonazza S, Nowik M, Peluso G, Massara A (2014). Body weight, gender and response to TNF-α blockers in axial spondyloarthritis. Rheumatology..

[29] Lubrano E, Perrotta FM, Manara M, D’Angelo S, Addimanda O, Ramonda R (2018). The sex influence on response to tumor necrosis factor-α inhibitors and remission in axial spondyloarthritis. J Rheumatol.

[30] Rusman T, van Vollenhoven RF, van der Horst-Bruinsma IE (2018). Gender differences in axial spondyloarthritis: women are not so lucky. Curr Rheumatol Rep.

