# Supplementary material for: Structural changes in the sacroiliac joint on MRI and relationship to ASDAS inactive disease in axial spondyloarthritis: a 2-year study comparing treatment with etanercept in EMBARK to a contemporary control cohort in DESIR
Source: Arthritis Res Ther. 2021 Jan 29;23:43. doi: 10.1186/s13075-021-02428-8 (PMC7844996; doi:10.1186/s13075-021-02428-8)
Supplement: Supplementary file 1 — Additional file 1: Table S1. Lesion change on MRI in patients with axial spondyloarthritis, baseline to Week 104; Figure S1. Cumulative probability of change in MRI structural lesion score in patients with axial spondyloarthritis for fat metaplasia (a) and ankylosis (b) over 104 weeks, average of the readers; Table S2. Pearson correlations between the baseline covariates; Table S3. Significant subset of predictors of Week 104 structural lesion change categories, from stepwise selection models (with predictors of study, sex, and Week 104 3-level ASDAS forced into model); Table S4. Significant subset of predictors of Week 104 structural lesion change categories, from stepwise selection models (with no forcing of predictors into model); Table S5. Decrease or increase in MRI structural lesions of erosion and backfill according to sustained ASDAS outcome in patients with axial spondyloarthritis, baseline to Week 104; Figure S2. Proportion of patients with axial spondyloarthritis with increase or decrease in fat metaplasia (a), and increase or decrease in ankylosis (b) according to ASDAS outcome, baseline to Week 104. [file 13075_2021_2428_MOESM1_ESM.pdf]

Additional file 1

Table S1 Lesion change on MRI in patients with axial spondyloarthritis, baseline to Week 104

				Unadjusted analysis		Adjusted analysis[a]	
Structural lesion	Study cohort	Lesion decreased n/N (%)	Lesion increased n/N (%)	Net % patients with decrease within study cohort (95% CI)	Net % patients with decrease (Etanercept ─ Control) (95% CI)	Net % patients with decrease within study cohort (95% CI)	Net % patients with decrease (Etanercept ─ Control) (95% CI)
Erosion	Etanercept (EMBARK)	46/163 (28.2)	7/163 (4.3)	23.9%*** (15.7, 32.2)	18.7%* (4.1, 33.3)	23.1%*** (13.2, 33.0)	20.2%* (4.6, 35.8)
	Control (DESIR)	14/76 (18.4)	10/76 (13.2)	5.3% (–6.8,17.3)		2.9% (–11.2, 16.9)	
Structural lesion	Study cohort	Lesion increased n/N (%)	Lesion decreased n/N (%)	Net % patients with increase within study cohort (95% CI)	Net % patients with increase (Etanercept ─ Control) (95% CI	Net % patients with increase within study cohort (95% CI)	Net % patients with increase (Etanercept ─ Control) (95% CI
Backfill	Etanercept (EMBARK)	27/163 (16.6)	1/163 (0.6)	16.0%*** (10.0, 21.9)	5.4% (–5.1, 15.9)	16.5%*** (9.2, 23.7)	5.4% (–6.0, 16.8)
	Control (DESIR)	10/76 (13.2)	2/76 (2.6)	10.5%* (1.8, 19.2)		11.0%* (0.8, 21.3)	
Fat metaplasia	Etanercept (EMBARK)	14/163 (8.6)	2/163 (1.2)	7.4%** (2.7, 12.0)	–1.8% (–10.1, 6.4)	9.2%** (3.7, 14.7)	3.9% (–4.8, 12.5)
	Control (DESIR)	7/76 (9.2)	0/76 (0)	9.2%** (2.4, 16.0)		5.3% (–2.5, 13.2)	
Ankylosis	Etanercept (EMBARK)	2/163 (1.2)	2/163 (1.2)	0% (–2.2, 2.2)	-1.3% (-5.3, 2.7)	–1.5% (–4.5, 1.4)	–1.2% (–5.9, 3.4)
	Control (DESIR)	1/76 (1.3)	0/76 (0)	1.3% (–2.0, 4.6)		–0.3% (–4.5, 3.9)	

[a]Adjusted for covariates at baseline: sex, symptom duration, smoking status, human leukocyte antigen-B27 status, ankylosing spondylitis disease activity score, Spondyloarthritis Research Consortium of Canada MRI Sacroiliac Joint (SIJ) inflammation score, erosion score (average of 3 readers), total SIJ score based on modified New York grade (average of 3 readers).

*$P < 0.05$, **$P < 0.01$, ***$P < 0.001$.

Fig. S1 Cumulative probability of change in MRI structural lesion score in patients with axial spondyloarthritis for fat metaplasia (**a**) and ankylosis (**b**) over 104 weeks, average of the readers.

a

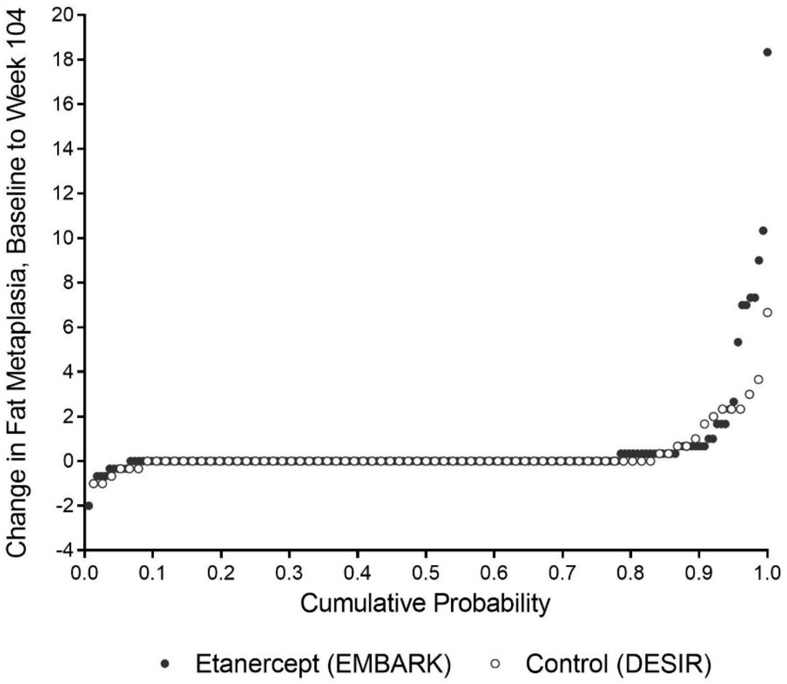

b

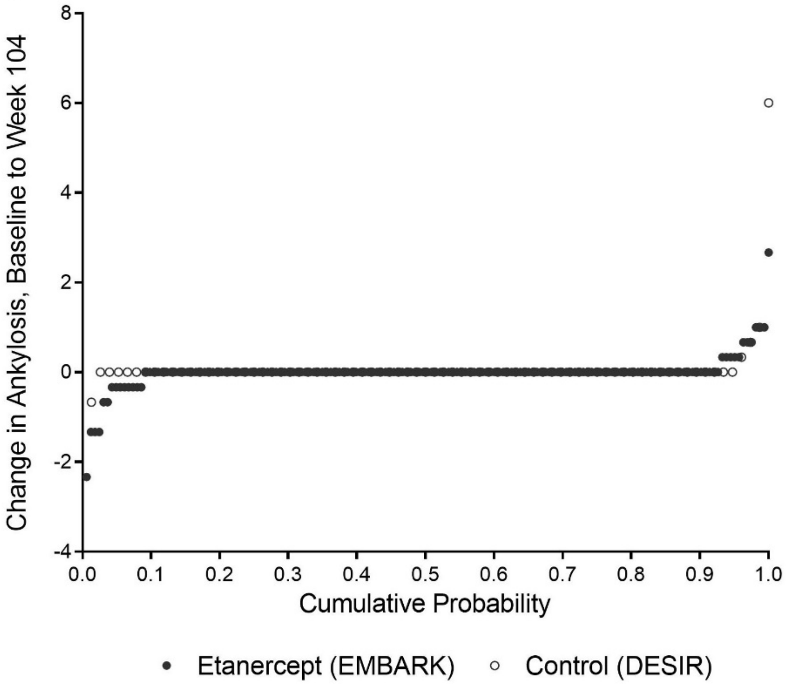

Table S2 Pearson correlations between the baseline covariates

Covariate	Symptom duration	ASDAS	SPARCC MRI SIJ inflammation score	Total SIJ score[a,b]
ASDAS	r = 0.08			
SPARCC MRI SIJ inflammation score	r = 0.02	r = 0.17*		
Total SIJ score[a,b]	r = 0.06	r = −0.05	r = 0.26**	
SSS erosion score[a]	r = 0.05	r = 0.02	r = 0.64**	r = 0.45**

[a]Average of the 3 readers.

[b]Based on the modified New York grading system.

*$P < 0.05$, **$P < 0.001$.

All patients had axial spondyloarthritis.

ASDAS ankylosing spondylitis disease activity score, *r* Pearson correlation, *SPARCC MRI SIJ inflammation score* Spondyloarthritis Research Consortium of Canada Magnetic Resonance Imaging Sacroiliac Joint score (a score of MRI bone marrow edema), *SSS* SIJ Structural Score.

Table S3 Significant subset of predictors of Week 104 structural lesion change categories, from stepwise selection models (with predictors of study, sex, and Week 104 3-level ASDAS forced into model)

Structural lesion	Significant predictor	Predictor category	Predictor estimate (SE)	P value
Erosion	Intercept	Intercept	-19.2 (8.5)	0.025
	Sex	Male	0	
		Female	3.5 (7.0)	0.62
	BL SSS erosion score[a]	BL SSS erosion score[a]	4.4 (1.4)	0.001
	BL SPARCC MRI SIJ inflammation score	BL SPARCC MRI SIJ inflammation score	1.8 (0.4)	<0.001
	Study	EMBARK	16.5 (7.3)	0.026
		DESIR	0	
	Sustained response	ASDAS <1.3	0	
		ASDAS >1.3 to <2.1	-0.4 (8.2)	0.96
		ASDAS ≥2.1	0.6 (8.6)	0.94
Backfill	Intercept	Intercept	1.0 (6.2)	0.87
	Sex	Male	0	
		Female	-2.5 (5.1)	0.63
	BL SSS erosion score[a]	BL SSS erosion score[a]	-2.8 (1.0)	0.005
	BL SPARCC MRI SIJ inflammation score	BL SPARCC MRI SIJ inflammation score	-1.2 (0.3)	<0.001
	Study	EMBARK	-3.4 (5.3)	0.52
		DESIR	0	
	Sustained response	ASDAS <1.3	0	
		ASDAS >1.3 to <2.1	12.4 (5.9)	0.037
		ASDAS ≥2.1	5.5 (6.2)	0.38
Fat metaplasia	Intercept	Intercept	3.9 (4.8)	0.42
	Sex	Male	0	
		Female	-1.8 (3.9)	0.64
	BL SSS erosion score[a]	BL SSS erosion score[a]	-2.6 (0.8)	0.002
		BL total SIJ score[a,b]	3.3 (1.5)	0.025

	BL SPARCC MRI SIJ inflammation score	BL SPARCC MRI SIJ inflammation score	-0.8 (0.2)	<0.001
	Study	EMBARK	-2.3 (4.1)	0.58
		DESIR	0	
	Sustained response	ASDAS <1.3	0	
		ASDAS >1.3 to <2.1	1.0 (4.6)	0.83
		ASDAS ≥2.1	-5.7 (4.8)	0.24
Ankylosis	Intercept	Intercept	3.1 (2.5)	0.22
	Sex	Male	0	
		Female	2.8 (1.8)	0.13
	BL SSS ankylosis score[a]	BL SSS ankylosis score[a]	4.6 (0.8)	<0.001
	Smoking	No	-4.6 (2.0)	0.027
		Yes	0	
	Study	EMBARK	-1.2 (2.0)	0.54
		DESIR	0	
	Sustained response	ASDAS <1.3	0	
		ASDAS >1.3 to <2.1	-0.3 (2.3)	0.90
		ASDAS ≥2.1	-4.7 (2.4)	0.0499

[a]Average of the 3 readers.

[b]Based on the modified New York grading system.

All patients had axial spondyloarthritis.

ASDAS ankylosing spondylitis disease activity score, *BL* baseline, *SE* standard error, *SPARCC MRI SIJ inflammation score* Spondyloarthritis Research Consortium of Canada Magnetic Resonance Imaging Sacroiliac Joint score (a score of MRI bone marrow edema), *SSS* SIJ Structural Score.

Table S4 Significant subset of predictors of Week 104 structural lesion change categories, from stepwise selection models (with no forcing of predictors into model)

Structural lesion	Significant predictor	Predictor category	Predictor estimate (SE)	P value
Erosion	Intercept	Intercept	-17.3 (6.2)	0.006
	BL SSS erosion score[a]	BL SSS erosion score[a]	4.3 (1.3)	0.001
	BL SPARCC MRI SIJ inflammation score	BL SPARCC MRI SIJ inflammation score	1.7 (0.4)	<0.001
	Study	EMBARK	16.2 (6.9)	0.020
		DESIR	0	
Backfill	Intercept	Intercept	1.9 (2.9)	0.52
	BL SSS erosion score[a]	BL SSS erosion score[a]	-2.7 (1.0)	0.005
	BL SPARCC MRI SIJ inflammation score	BL SPARCC MRI SIJ inflammation score	-1.2 (0.3)	<0.001
Fat metaplasia	Intercept	Intercept	0.5 (2.7)	0.84
	BL SSS erosion score[a]	BL SSS erosion score[a]	-2.5 (0.8)	0.002
		BL total SIJ score[a,b]	3.0 (1.4)	0.035
	BL SPARCC MRI SIJ inflammation score	BL SPARCC MRI SIJ inflammation score	-0.8 (0.2)	<0.001
Ankylosis	Intercept	Intercept	-1.2 (0.9)	0.18
	BL SSS ankylosis score[a]	BL SSS ankylosis score[a]	4.8 (0.8)	<0.001

[a]Average of the 3 readers.

[b]Based on the modified New York grading system.

All patients had axial spondyloarthritis.

ASDAS ankylosing spondylitis disease activity score, *BL* baseline, *SE* standard error, *SPARCC MRI SIJ inflammation score* Spondyloarthritis Research Consortium of Canada Magnetic Resonance Imaging Sacroiliac Joint score (a score of MRI bone marrow edema), *SSS* SIJ Structural Score.

Table S5 Decrease or increase in MRI structural lesions of erosion and backfill according to sustained ASDAS outcome in patients with axial spondyloarthritis, baseline to Week 104

				Unadjusted analysis			Adjusted analysis[a]		
**Erosion**									
Sustained ASDAS Outcome	Study cohort	Lesion decreased n/N (%)	Lesion increased n/N (%)	Net % patients with decrease within study cohort (95% CI)	*P* value for trend across ASDAS categories (both studies pooled)	*P* value for difference between studies	Net % patients with decrease within study cohort (95% CI)	*P* value for trend across ASDAS categories (both studies pooled)	*P* value for difference between studies
ASDAS < 1.3	Etanercept (EMBARK)	34/104 (32.7)	5/104 (4.8)	27.9%*** (17.5, 38.3)	0.28	0.06	20.9%*** (8.8, 33.0)	0.97	0.01
	Control (DESIR)	5/23 (21.7)	2/23 (8.7)	13.0% (–9.0, 35.1)			5.9% (–17.9, 29.8)		
ASDAS ≥1.3 to <2.1	Etanercept (EMBARK)	7/33 (21.2)	1/33 (3.0)	18.2% (–0.3, 36.6)			25.8%** (6.9, 44.7)		
	Control (DESIR)	4/24 (16.7)	4/24 (16.7)	0% (–21.6, 21.6)			3.4% (–19.8, 26.6)		
ASDAS ≥ 2.1	Etanercept (EMBARK)	5/24 (20.8)	1/24 (4.2)	16.7% (–5.0, 38.4)			28.7%** (7.8, 49.6)		
	Control (DESIR)	5/29 (17.2)	4/29 (13.8)	3.4% (–16.2, 23.1)			–1.2% (–20.9, 18.5)		
**Backfill**									
Sustained ASDAS Outcome	Study cohort	Lesion increased n/N (%)	Lesion decreased n/N (%)	Net % patients with increase within study cohort (95% CI)	*P* value for trend across ASDAS categories (both studies pooled)	*P* value for difference between studies	Net % patients with increase within study cohort (95% CI)	*P* value for trend across ASDAS categories (both studies pooled)	*P* value for difference between studies

ASDAS < 1.3	Etanercept (EMBARK)	23/104 (22.1)	0/104 (0)	22.1%*** (14.8, 29.5)	0.03	0.99	20.4%*** (11.7, 29.2)	0.15	0.85
	Control (DESIR)	5/23 (21.7)	0/23 (0)	21.7%** (6.1, 37.4)			23.2%** (6.0, 40.5)		
ASDAS ≥1.3 to <2.1	Etanercept (EMBARK)	2/33 (6.1)	0/33 (0)	6.1% (–7.0, 19.1)			10.0% (–3.6, 23.7)		
	Control (DESIR)	1/24 (4.2)	1/24 (4.2)	0% (–15.3, 15.3)			5.5% (–11.3, 22.3)		
ASDAS ≥ 2.1	Etanercept (EMBARK)	2/24 (8.3)	1/24 (4.2)	4.2% (–11.1, 19.5)			12.7% (–2.4, 27.8)		
	Control (DESIR)	4/29 (13.8)	1/29 (3.4)	10.3% (–3.6, 24.3)			10.7% (–3.6, 24.9)		

[a]Adjusted for covariates at baseline: sex, symptom duration, smoking status, human leukocyte antigen-B27 status, ankylosing spondylitis disease activity score (ASDAS), Spondyloarthritis Research Consortium of Canada MRI Sacroiliac Joint (SIJ) inflammation score, erosion score (average of 3 readers), and total SIJ score based on modified New York grade (average of 3 readers).

*$P < 0.05$, **$P < 0.01$, ***$P < 0.001$ for decrease vs increase within ASDAS category.

Fig. S2 Proportion of patients with axial spondyloarthritis with increase or decrease in fat metaplasia (**a**), and increase or decrease in ankylosis (**b**) according to ASDAS outcome, baseline to Week 104.

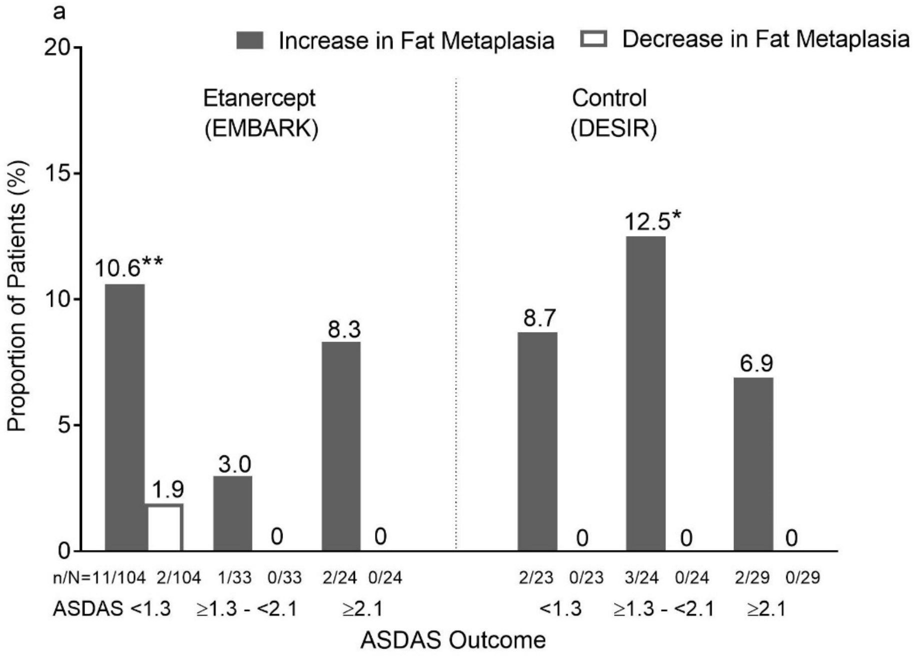

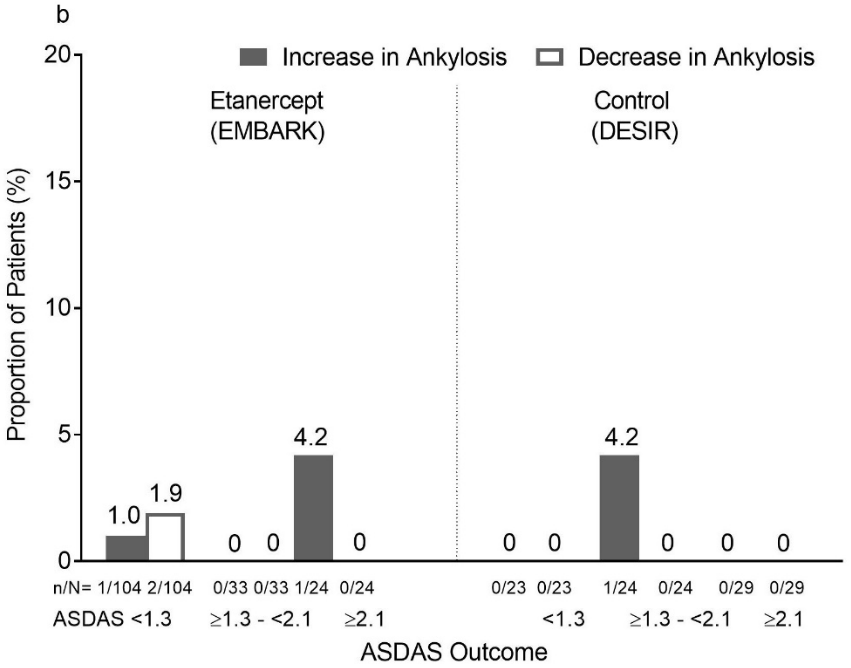

**P*<0.05, ***P*=0.01 for increase vs decrease within ASDAS category, unadjusted

ASDAS ankylosing spondylitis disease activity score.
